# Modulation of Crystallinity through Radiofrequency Electromagnetic Fields in PLLA/Magnetic Nanoparticles Composites: A Proof of Concept

**DOI:** 10.3390/ma14154300

**Published:** 2021-07-31

**Authors:** Marta Multigner, Irene Morales, Marta Muñoz, Victoria Bonache, Fernando Giacomone, Patricia de la Presa, Rosario Benavente, Belén Torres, Diego Mantovani, Joaquín Rams

**Affiliations:** 1 Department of Applied Mathematics, Materials Science and Engineering and Electronic Technology, Universidad Rey Juan Carlos, 28933 Madrid, Spain; marta.munoz@urjc.es (M.M.); victoria.bonache@urjc.es (V.B.); belen.torres@urjc.es (B.T.); joaquin.rams@urjc.es (J.R.); 2 Instituto de Magnetismo Aplicado, UCM-ADIF-CSIC, A6 22,500 Km, 28230 Las Rozas, Spain; irenemorales@ucm.es (I.M.); giacomone@gmx.es (F.G.); pmpresa@ucm.es (P.d.l.P.); 3 Institute of Polymer Science and Technology (ICTP-CSIC), C/Juan de la Cierva 3, 28006 Madrid, Spain; rbenavente@ictp.csic.es; 4 Laboratory for Biomaterials & Bioengineering (CRC-I), Department Min-Met-Materials Engineering & Research Center CHU de Québec, Laval University, Québec, QC G1V 0A6, Canada; diego.mantovani@gmn.ulaval.ca

**Keywords:** biodegradable nanocomposite, PLLA, magnetic nanoparticles, radiofrequency electromagnetic field

## Abstract

To modulate the properties of degradable implants from outside of the human body represents a major challenge in the field of biomaterials. Polylactic acid is one of the most used polymers in biomedical applications, but it tends to lose its mechanical properties too quickly during degradation. In the present study, a way to reinforce poly-L lactic acid (PLLA) with magnetic nanoparticles (MNPs) that have the capacity to heat under radiofrequency electromagnetic fields (EMF) is proposed. As mechanical and degradation properties are related to the crystallinity of PLLA, the aim of the work was to explore the possibility of modifying the structure of the polymer through the heating of the reinforcing MNPs by EMF within the biological limit range *f*·*H* < 5·× 10^9^ Am^−1^·s^−1^. Composites were prepared by dispersing MNPs under sonication in a solution of PLLA. The heat released by the MNPs was monitored by an infrared camera and changes in the polymer were analyzed with differential scanning calorimetry and nanoindentation techniques. The crystallinity, hardness, and elastic modulus of nanocomposites increase with EMF treatment.

## 1. Introduction

One of the challenges of degradable biomaterials is to synchronize the biodegradation process of the implant and the regeneration time of living tissues. In the specific case of bone repair, bioabsorbable polymeric materials such as polylactic acid and copolymers are currently used [1]. Depending on the proportion of each of its two enantiomers, L or D, the glass transition temperatures can vary between 50 and 65 °C, as well as other thermal properties such as crystallization ability, and consequently, the mechanical properties and degradation rate [2,3].

On the other hand, magnetic nanoparticles (MNPs) possess the capability to dissipate energy and to heat the surrounding media when subjected to radiofrequency electromagnetic fields. In fact, they have been widely studied for more than 20 years as an anti-tumor treatment known as hyperthermia, which consists of heating the cancerous tissues to temperatures between 41 and 46 °C [4]. The energy dissipation tends to increase as the field frequency and amplitude increase. However, for biomedical reasons, there is a limit to the frequency and amplitude of the applied field; that is, the product *f*·*H* must be smaller than 5 × 10^9^ Am^−1^·s^−1^ [5,6]. Furthermore, from a clinical point of view, although MNPs such as γ-Fe_2_O_3_ and Fe_3_O_4_ present excellent biocompatibility, the objective is to incorporate them into the body to the minimum the quantity of foreign substance. Therefore, huge efforts have been undertaken to obtain MNPs with high heating efficiency. The heating efficiency depends on:

1. Intrinsic properties of MNPs, i.e., particle size and shape, saturation magnetization, anisotropy field, and aggregation degree [7,8,9,10,11,12];
2. Extrinsic conditions, such as frequency and amplitude of the magnetic field and media viscosity [10,11,12,13,14].

The amount of heat released by the MNPs depends on the area of the hysteresis cycle under EMF and the frequency of the field [7]. The magnetization reversal is governed by two mechanisms: one associated with the physical movement of the whole MNPs, known as the Brown relaxation process; and another one associated with the rotation of the magnetic moments inside the MNPs, known as the Néel relaxation process. For a given magnetic field amplitude and frequency, the viscosity of the medium plays a significant role in the heating efficiency, depending on the relaxation process that predominates. For low viscosity media, both mechanisms can be present depending on particle size, magnetization and anisotropy field of the MNPs. However, for high viscosity media (at the limit, solids), only the Néel relaxation mechanism is expected to play a decisive role in the heating process [14]. It is not clear yet to what extent the disappearance of one of the two mechanisms will affect the heating efficiency when the MNPs are embedded in a solid material.

The aim of this research was to explore the changes of a solid biodegradable polymer, poly-L-lactic acid (PLLA), induced by local heating of MNPs and electromagnetic fields within the biological limit range *f*·*H* < 5 × 10^9^ Am^−1^·s^−1^.

For this exploratory work, two different MNPs, γ-Fe_2_O_3_ and MnFe_2_O_4_, were selected for reinforcing PLLA. It has been previously proved that both MNPs can achieve specific absorption rate (SAR) values of about 40–100 W/g in different colloidal systems [12,15,16]. The composite material was exposed to electromagnetic fields of different frequencies and intensities to heat and modify the crystallization state of the polymer. It is well known that the crystallinity and the size and compaction of the crystallites affects both the rate of degradation and the mechanical properties of polymers [4,15,16].

It can be difficult to predict whether the local heating transmission from MNPs to the polymer matrix can cause significant changes in a macroscopic scale. If that happens, since the human body is transparent to electromagnetic fields, it could be possible to modulate the mechanical properties and biodegradation rate of implants non-invasively from outside of the body by using electromagnetic fields that are harmless to the patient.

## 2. Materials and Methods

The MnFe_2_O_4_ MNPs were synthesized by co-precipitation with an average diameter of 11.7 ± 0.3 nm. Details about the synthesis procedure are reported elsewhere [15]. Briefly, under air atmosphere, 2.5 mmol of MnCl_2_·4H_2_O was dissolved in 250 μL of HCl 37%, and 4 mL of water was added. On the other hand, 5 mmol of FeCl_3_·6H_2_O was dissolved in 10 mL of water. Both solutions were heated at 50 °C, mixed, and added to a solution of 50 mL NaOH (3 M) at 100 °C. The stirring was adjusted to 600 rpm, and a black precipitate formed immediately. The synthesis temperature was kept constant at 100 °C for 30 min. After that, the mixture was cooled down to room temperature and magnetically separated and washed several times with distilled water by magnetic decantation. Finally, the precipitate was dispersed in 0.1 M TMAOH (tetramethylammonium hydroxide).

γ-Fe_2_O_3_ MNPs were obtained by following a modified Massart coprecipitation protocol as reported in [14]. Briefly, Fe_3_O_4_ nanoparticles were synthesized by adding 425 mL of an aqueous solution of FeCl_3_·6H_2_O (0.09 mol) and FeCl_2_·4H_2_O (0.054 mol) to 75 mL of an alkaline medium. The synthesis was carried out under air atmosphere. The particle size can be tuned by the nature of the alkaline medium, the addition rate, and the aging time. Slow addition rates (0.2 mL/s) over NH_4_OH (25%), followed by a heating process at 90 °C for 3 h were used. After synthesis, the particles were washed three times with distilled water at 25 °C and collected with the help of a permanent magnet.

A standard protocol was used to oxidize magnetite to maghemite [17]. In addition, this treatment activates the particle surface for further coating with aminopropylsilane (APS) groups. Briefly, 300 mL of HNO_3_ (2 M) (Sigma Aldrich, St. Louis, MO, USA) was added to 500 mL of the dispersion produced previously, and the mixture was stirred for 15 min. Then, the supernatant was removed by magnetic decantation and 75 mL of Fe(NO_3_)_3_ (1 M) and 130 mL of water were added. The mixture was heated to boiling temperature and stirred for 30 min. The particles were then cooled to room temperature and, by magnetic decantation, the supernatant was substituted with 300 mL of HNO_3_ (2 M), and the solution was stirred for 15 min. Finally, the particles were washed three times with acetone and dispersed in water. A rotary evaporator was used to remove any acetone waste as well as to concentrate the sample.

The size and shape of the particles were measured by transmission electron microscopy (TEM) using a JEOL-2000FXII (JEOL, Tokyo, Japan) at 200 kV. The mean particle size and distribution were calculated by measuring more than 100 particles using Digital Micrograph™. The experimental data were fitted to a lognormal distribution to obtain the mean particle size (*d*) and standard deviation (σ). Magnetic properties of MNPs have already been published [14]. MNPs suspension was dried at 40 °C for 24 h to obtain a powder to be dispersed on PLLA. The powder was later milled in agate mortar until a homogeneous fine powder was obtained.

Commercially available PLLA (Goodfellow) was used as polymeric matrix. PLLA particles with 3 mm of nominal granule size were dissolved (6 wt%) in analytical grade chloroform (Sigma Aldrich, St. Louis, USA) by magnetic stirring at 25 °C. MNPs were incorporated at different concentrations and dispersed using a UP400S (400 W, 24 kHz)-(Hielscher Utrasonics GmbH, Teltow, Germany) sonication equipment for 10 min. Subsequently, the suspension was cast in a 10 cm diameter mold. Films of about 0.3 mm thick were obtained. Samples were aged in an extractor hood at 25 °C for 7 days to facilitate the evaporation of chloroform. After that, all the samples were stored together for the same time at 25 °C in close recipients. Circles 2 cm in diameter were extracted from films for further treatment with electromagnetic fields.

The nanocomposite samples in this study are referred to as PLLA, followed by the weight percentage value and “Fe” for γ-Fe_2_O_3_ or “Mn” for MnFe_2_O_4_ MNPs. Thus, for instance, PLLA13Fe stands for PLLA/MNP nanocomposite with 13 wt% of γ-Fe_2_O_3_ MNPs.

Finally, the mass concentration of MNPs in the composites was determined by thermogravimetric analysis, using TGA/SDTA 851e (Mettler Toledo, Toronto, Canada) equipment. About 1 mg of sample was put into a 70 μL alumina crucible and then heated from 25 °C to 800 °C with a heating rate of 10 °C/min. A protective atmosphere of N_2_ gas was used.

The heating capabilities of MNPs were measured with the commercial system Magnetherm 1.5 (Nanotherics, Warrington, UK). The mean magnetic field inside the coil were obtained by measuring the induced voltage in a two turns secondary coil with a cross-sectional value close to that of the sample holder (6.2 mm diameter) and solving Faraday’s Law. The sample was placed to coincide with the maximum value of the magnetic field. The temperatures of the coils were controlled through a closed circuit of water maintained at 16 °C with a cryostat bath.

Electromagnetic field (EMF) treatments were applied to the composites with the Magnetherm equipment. Two different frequencies were chosen, 112 and 331 kHz, with the maximum amplitude available for the equipment of 18.5 and 10.1 kAm^−1^, respectively. The sample holder, an open squared plastic box, was placed inside the coil with the 20 mm diameter sample at the center.

The EMF heating effect was measured in both (i) the colloid suspension of MNPs in water and (ii) the MNPs embedded in solid PLLA/MNPs composite films. The colloid temperature was measured with an optical fiber thermometer and recorded with a computer. The temperature of PLLA/MNPs composite films was measured with an infrared camera FLIR E50. The following methodology was followed for both kinds of measurements: prior to turning the magnetic field on, the sample temperature was recorded for about 30 s to ensure thermal stability and to have a baseline for the calculation of the SAR. As the field was turned on, the temperature increase was measured for 300 s. By performing a linear fit of the data (temperature versus time) in the initial time interval, the slope Δ*T*/Δ*t* could be obtained. The time range was selected in the linear part of the heating curve (Figures S1 and S2) in the first 20 s right after turning the field ON. It is worth noting that although the coils increased their temperature during treatment, the space between the sample and the coil was colder than any of them, ensuring that the temperature increase was coming from the samples and not from the coils. Temperature was registered at three points along the radius of the sample.

Differential scanning calorimetry (DSC) measurements were performed with DSC 823e (Mettler Toledo, Toronto, Canada) equipment. Temperature and heat flow were calibrated in N_2_ atmosphere with an indium calibration standard. Samples were heated at a 10 °C/min rate between 25 and 200 °C. Enthalpy of melting, ∆*H_m_*, and cold crystallization, ∆*H_cc_*, were obtained from the curves. The percentage of crystallinity was calculated using Equation (1), where ∆*H_mo_* (93.1 J/g) is the enthalpy of 100% crystalline PLLA [18].

*χ* = (∆*H_m_* − ∆*H_cc_*)/∆*H_mo_* (1)

Fourier transform infrared spectroscopy (FTIR) spectra of the samples were recorded by performing 64 scans in a Fourier transform infrared spectrometer (FT-IR Varian Excalibur 3100, Varian Medical Systems, Alcobendas, Spain) in the 3500–600 cm^−1^ range in ATR, at a resolution of 4 cm^−1^ and with four measurements for every sample. A separate background spectrum was subtracted in each collection.

The morphology of composites was observed with a Hitachi S-3400N scanning electron microscope (SEM) (Hitachi High-Tech, Krefeld, Germany) equipped with an energy dispersive X-ray spectrometer (EDX).

The mechanical properties, in terms of hardness and Young’s modulus, were evaluated by the nanoindentation technique. For this purpose, the cross section of samples was grounded and polished with 0.25 μm Al_2_O_3_ suspension.

The tests were performed in an XP Nanoindenter (MTS, Eden Prairie, USA) with a pyramidal Berkovich tip working in continuous stiffness measurement (CSM) mode. In this method, hardness and modulus profiles are obtained during loading, which provides high accuracy and resolution, also improving the surface detection.

At least 25 indentation tests, until a penetration depth of 1000 nm, using 5 nm harmonic oscillation amplitude, were performed for each material, in order to obtain representative results. To analyze the distribution of properties in the material, in some composites, matrices of 100–200 (10 × 10–20 × 10) tests at 500 nm penetration and with 10 μm spacings were performed. The location of each indentation was subsequently verified by optical microscopy.

## 3. Results and Discussion

### 3.1. Structure of MNPs

**Figure 1:**
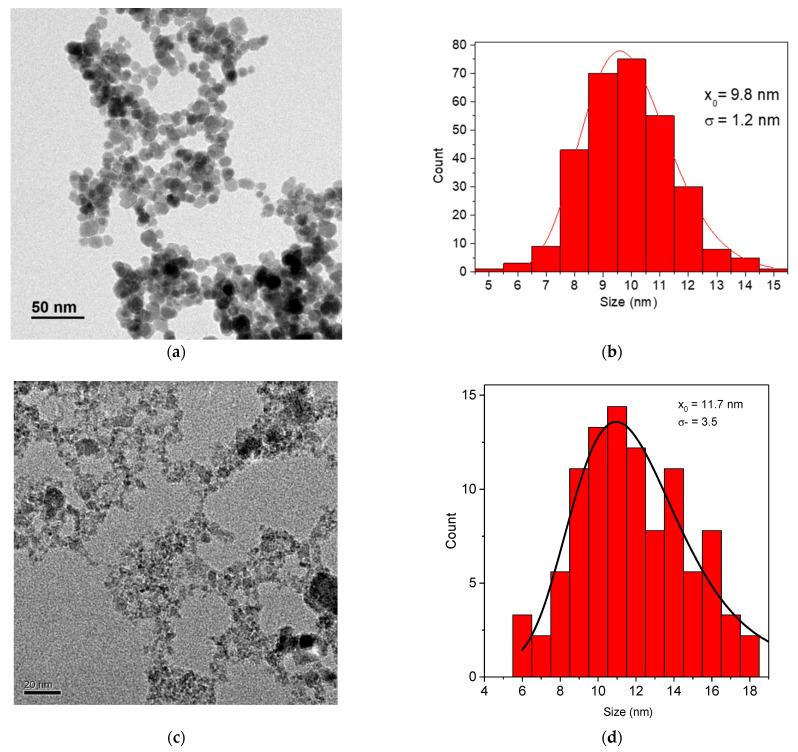
TEM image (**a**) and size distribution (**b**) of γ-Fe_2_O_3_ NPs and TEM image (**c**) and size distribution (**d**) of MnFe_2_O_4_ NPs. (Figure 1c,d reprinted with permission from ref. [15]. Copyright 2016 Elsevier).

Figure 1a shows a TEM image and size distribution of γ-Fe_2_O_3_ NPs, which have an average particle size of 9.8 nm with a low dispersity degree of 0.12 (standard deviation/mean size). MnFe_2_O_4_ NPs have an average size of 11.7 nm and dispersity degree of 0.3 [17] (Figure 1b).

### 3.2. Morphology

**Figure 2:**
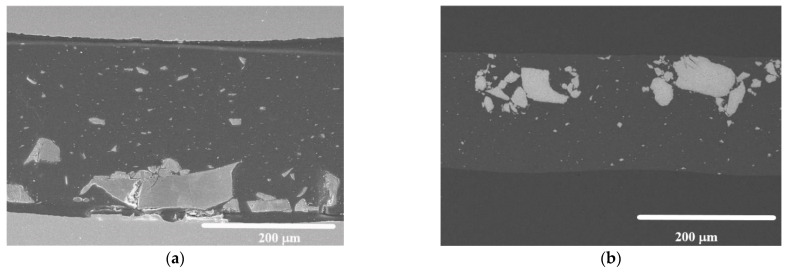
Cross section SEM micrographs. (**a**) As-prepared PLLA13Fe (secondary electron image); (**b**) as prepared PLLA9Mn (back-scattered electron image, BSE).

To the naked eye, the composite samples presented a homogenous aspect with brown or grey/black color, depending on the type of MNPs, MnFe_2_O_4_ or γ-Fe_2_O_3_, respectively, and on their concentration. However, SEM micrographs revealed an inhomogeneous distribution of the particles inside the PLLA matrix and powder particle aggregates up to 200 μm were observed (Figure 2). The thickness of the films was continuous and no defects were observed in them.

### 3.3. Thermogravimetry

**Figure 3:**
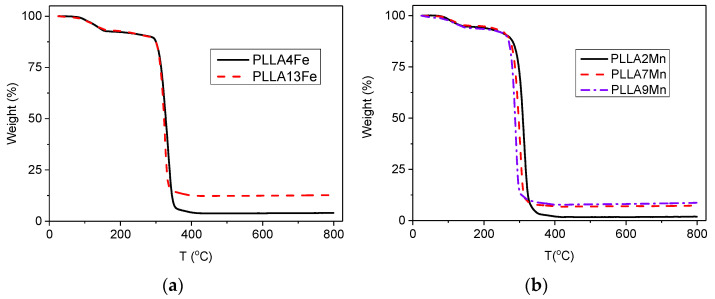
TG thermograms of (**a**) PLLA/γ-Fe_2_O_3_ and (**b**) PLLA/MnFe_2_O_3_ composites.

TG measurements are shown in Figure 3. A shoulder between 100 and 150 °C can be appreciated with a mass reduction of about 6%. Most probably, this was due to evaporation of chloroform (Tb = 61 °C) or water still trapped in the structure of PLLA. Above 300 °C, the organic compound is completely carbonized and the MNPs content can be determined as the remnant mass. Table 1 summarizes the measured mass percentages of MNPs in the PLLA matrix.

The temperature of maximum weight loss rate (*T_p_*) (Table 1), calculated from the derivative of the curves, tends to diminish with increasing MNPs content, which implies that the energy required for the PLLA degradation reduces as MNPs concentration increases. Similar behavior has been observed in Mg/PLLA composites [19] and has been attributed to a higher amount of adsorbed and absorbed water on the surface of the particles [20].

### 3.4. Heating Efficiency

The heating efficiency of MNPs suspended in water and subjected to the radiofrequency electromagnetic field are summarized in Table 2. SAR is calculated from the expression [21]

SAR = (*C_water_*/*c_NPs_*) (Δ*T*/Δ*t*) (2)

where *C_water_* is the specific heat capacity of water, 4.17 J/gK, and *c_NPs_* is the NPs weight concentration in the colloid. Table 2 shows SAR and d*T*/d*t* at the maximum applied field.

The highest SAR (Table 2) was achieved for MnFe_2_O_4_ NPs at 112 kHz and 18.5 kA/m^−1^. Differences in SAR values were related to the relaxation times of Néel and Brown mechanisms, *τ_N_* and *τ_B_*, respectively, and the influence of each mechanism in the heating process. As has been previously reported, both relaxation mechanisms, Néel and Brown, are present in the MnFe_2_O_4_ with a size of around 12 nm due to the broad particle size distribution and the magnetic properties of the material [15]. On the other hand, in the case of γ-Fe_2_O_3_ with a size of around 10 nm, the main relaxation process takes place by Néel mechanism [13,17].

**Figure 4:**
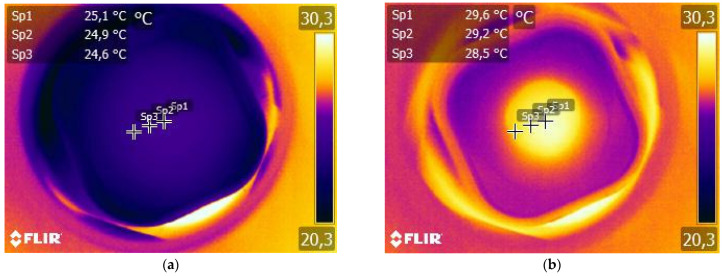
Infrared thermal image of PLLA13Fe composite sample before (**a**) and during (**b**) EM field treatment with 331 kHz and 10.1 kAm^−1^.

Figure 4 shows an example of an IR-camera image of the PLLA13Fe composite sample before (a) and during (b) the EMF treatment. Three points of the sample were chosen to measure the evolution of the temperature during the EMF treatment. Sp1 is placed at the center of the sample and Sp2 and Sp3 are progressively farther from the center. In Figure 4a, the geometry of the sample cannot be distinguished, as there are no significant differences in temperature between the sample and its surroundings when the field is not applied. However, when the EMF is on (Figure 4b), it is easy to recognize the circular composite sample, as it is hotter than its surroundings. The results are summarized in Table 3.

The sample that reached the highest SAR was the composite with MnFe_2_O_4_ NPs, which also achieved the highest values in the aqueous colloid state. Even though MNPs mass concentration is 77% higher in the PLLA than in water, SAR is almost 27 times smaller (see Table 2 and Table 3). There are some reasons that could explain this great difference:

1- The inhomogeneous distribution of the MNPs inside PLLA. It has been observed (Figure 2) that MNPs form aggregates of up to 200 μm. This, in turn, has two consequences:
  (a) Powder particles were obtained by drying a colloid where the MNPs tend to have a hydrodynamic radius of about some tens of nm [13,17]. During the drying process, the MNPs aggregate. In MNPs systems with high concentrations, the interparticle dipolar interaction becomes non-negligible and, depending on the strength of the interaction, they can even develop into a superspin glass or enter in a superferromagnetic state, where the collective behavior creates a condition of higher effective anisotropy [22,23,24]. This means that inside the aggregate a stronger field is needed to magnetize the MNPs and, consequently, to also produce the same heat as in the disaggregated MNPs system.
  (b) The inhomogeneous distribution of particles in the PLL/MNPs yields regions with a high concentration of MNPs, forming aggregates that reach lower temperatures than the well-dispersed ones and others without MNPs. During the first seconds of the EMF treatment, this leads to regions of a few microns that increase their temperature while others are still cold; the infrared camera is not able to resolve this temperature distribution, and consequently, the average temperature of the sample could be smaller than in the case of the homogeneous particle distribution.

Therefore, aggregates with higher effective magnetic anisotropy than noninteracting MNPs, in addition to areas with MNP concentration depletion, would have generated a system where the energy transfer from MNPs to polymeric matrix would have been less efficient than in the colloidal system.

2- It is worth noticing that if the MNPs are immobilized in the composites, then the Brownian relaxation mechanism is not expected to occur. It has been previously demonstrated that for γ-Fe_2_O_3_ colloids with a particle size below 11 nm, the Brownian contribution to SAR is negligible [13]. In this case, the immobilization of the particles should not cause relevant effects. However, MnFe_2_O_4_ NPs with a size of about 12 nm dispersed in water contribute to heating by both the Néel and Brown mechanisms [17]. Therefore, its dispersion in a solid matrix will suppress the latest mentioned source of heating.

Regarding the properties of the matrix, the thermal conductivity of PLLA is lower than that of water (0.19 W/mK and 0.6 W/mK, respectively), but the heat capacity is also lower in the case of PLLA (1.8 J/gK and 4.18 J/gK, respectively) [25,26]. Then, there is not a clear tendency for explaining how the thermal properties of the matrix could influence the different observed behavior in colloid and composite.

Despite all the questions that are still open, and which would need further studies to address, one result is inferred from the experiments: all the composite samples increase their temperature during EMF treatment, although in none of them does the average temperature measured in the samples overcome 36 °C.

### 3.5. FTIR

**Figure 5:**
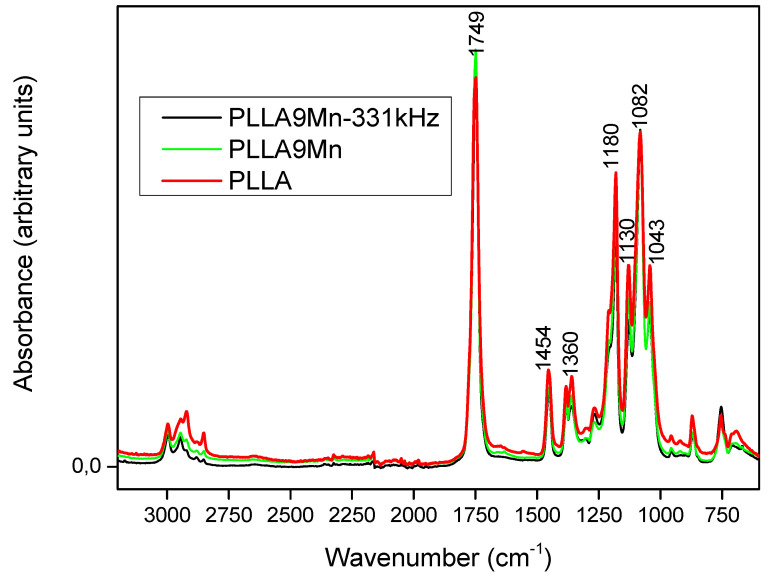
FTIR spectra of PLLA (red), PLLA9Mn (green), and PLLA9Mn treated with 331 kHz electromagnetic fields (black).

The chemical structure of PLLA with different MNP concentrations was studied by FTIR. Figure 5 shows the FTIR spectra of PLLA, PLLA9Mn, and PLLA9Mn treated with 331 kHz electromagnetic fields, with emphasis on the hydroxyl (-OH), methyl (C-H), and carbonyl (C=O) region. The most representative absorption bands of PLLA functional groups appear in the range of 800 to 1800 cm^−1^ for unsaturated groups, 1680 to 1800 cm^−1^ for the carbonyl group, and 2900 to 3000 cm^−1^ for CH_2_ and CH_3_ groups (Table S1).

MNPs did not alter the position of the strong band at 1749 cm^−1^, which is assigned to the characteristic absorption of carbonyl bond stretching vibration. The hydroxyl peak (>3000 cm^−1^) is absent in all the spectra. It is not possible to observe any molecular change in the chemical structure of the PLLA. Only a tiny change in the shape of the 2920 cm^−1^ band corresponded to the saturated region with no significance. There are no relevant changes related to the MNP content. It can be concluded that the PLLA chemical structure is not generally altered with the incorporation of MNPs or with the EM field treatment.

### 3.6. Calorimetry

**Figure 6:**
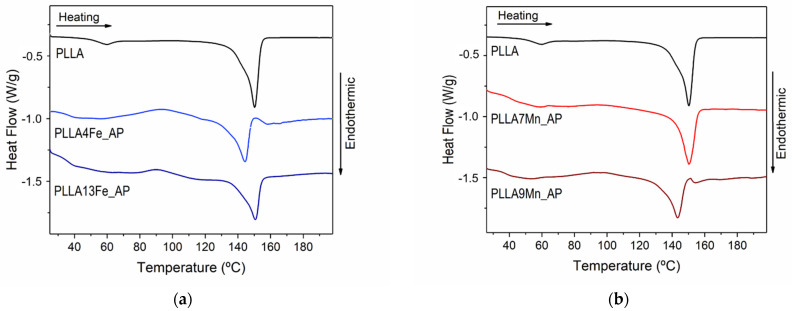
DSC scans of (**a**) PLLA and PLLA reinforced with 4.1 and 12.7 wt% of γ-Fe_2_O_3_ MNPs and (**b**) PLLA and PLLA reinforced with 7.4 and 8.7 MnFe_2_O_3_ MNPs.

**Figure 7:**
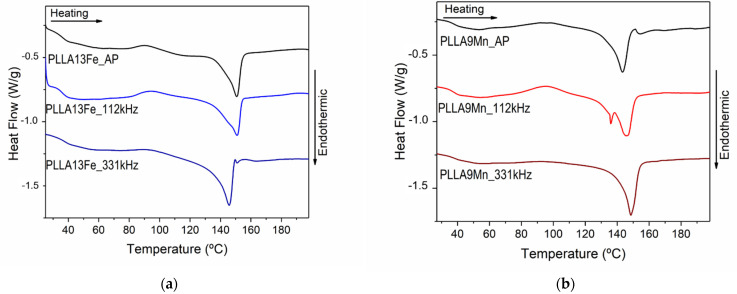
DSC scans of composites before and after EMF treatments at 112 and 331 KHz: (**a**) PLLA reinforced with 12.7 wt% of γ-Fe_2_O_3_ MNPs and (**b**) PLLA reinforced with 8.7 wt% of MnFe_2_O_3_ MNPs.

DSC thermograms were performed for pure PLLA as prepared PLLA/MNPs composites (AP) and EM field-treated PLLA/MNPs composites. Figure 6 shows the first heating DSC curves of pure PLLA, prepared under the same conditions as the composites, and of AP composites with different concentrations of MNPs. In Figure 7, the DSC scans of composites before and after the EM field treatment can be compared, and the data obtained from DSC measurements are summarized in Table 4.

As can be appreciated, the addition of MNPs reduces the cold crystallization temperature (*T*_cc_) and the glass transition temperature (*T*_g_) of the matrix. However, this latter effect is difficult to affirm soundly because the trapped chloroform has a plasticization effect that is revealed at the same temperature range (below 60 °C). Likewise, the presence of MNPs inhibits the PLLA crystallization, as can be inferred from the crystalline fraction data (see Table 4). In the PLLA4Fe_AP and PLLA9Mn_AP samples, two endothermic peaks corresponding to two melting temperatures appear, suggesting that the two crystalline phases nucleated. This finding can be attributed to two different crystallite size distributions, where the smaller ones have lower *T*_m_, or even to the presence of the less ordered α’ form together with the more ordered α form, which is the more common and stable form when PLA is obtained under conventional solution crystallization conditions. The first melting peak has been attributed to the simultaneous melting of the present α’ crystal form together with the recrystallization of the α’ into α crystal form, while during the higher temperature peak, the melting of the latter α crystal form developed during the recrystallization process takes place [27].

Regarding the effect of the EM field treatment, no temperatures were measured to be above the glass transition of the as-prepared samples (35–41 °C) (Table 3). However, the changes in the DSC curves (Figure 7) and the parameters derived from them (Table 4) reveal that the PLLA matrix, or part of it, of most of the composites, achieve temperatures above *T*_g_.

For low MNPs concentration, PLLA4Fe and PLLA7Mn, the EMF treatment did not affect the matrix (changes on *f*_c_ are within the experimental error).

The treatment with 112 kHz EM field of the PLLA13Fe composite leads to a lower crystalline fraction than in the as-prepared (AP) state. Whereas, with 331 kHz, *T*_m1_ shifts to lower temperatures and no changes in *T*_cc_ are detected. This can be due to a thickening of the more instable crystals (α’) that melt at lower *T*_m1_ than the AP (“as-prepared”) and 112 kHz composites. The PLLA9Mn composite treated at 112 kHz shows a DSC curve with a strongly bimodal behavior. The zones where the local heating is higher probably achieve a microstructure that helps to develop more stable α crystallites during the cold crystallization process with two different average crystallite sizes.

In the case of PLLA9Mn, the increase in the crystalline fraction from 20% in the as-prepared state to 33% for the one treated at 331 kHz and the disappearance of *T*_m1_ suggests that the dissipated energy helps to stabilize the smaller or less perfect crystals.

Even though a tendency that relates the measured temperature with the microstructure is not clearly defined, mainly due to the inhomogeneity of the powder particles dispersion and by the low volumes affected, it is evident that for the composites with high MNP concentration, the EMF treatment of the MNPs alters the microstructure of the PLLA matrix f_c_ and the crystallites size distribution. Although the infrared camera shows global temperature increases in the samples that keep below *T*g, high local heating is taking place with the EMF treatment, as is proved by changes in the DSC curves. A plausible explanation could be that the MNPs heat the PLLA that surrounds them, but its low thermal conductivity limits the heat propagation. In the future, to systematically study the relationship between the magnetic and heating properties under EMF and their effect on the crystallinity of PLLA, it would be necessary to develop a system that allows fabrication of the composite with the reinforcing phase homogeneously distributed.

### 3.7. Mechanical Characterization

PLLA4Fe, PLLA13Fe, and PLLA9Mn samples as prepared and as treated with the EM field that causes the highest temperature increase for each case (331 kHz for PLLA4Fe and PLLA13Fe and 112 kHz for PLLA9Mn) were characterized by the nanoindentation technique.

**Figure 8:**
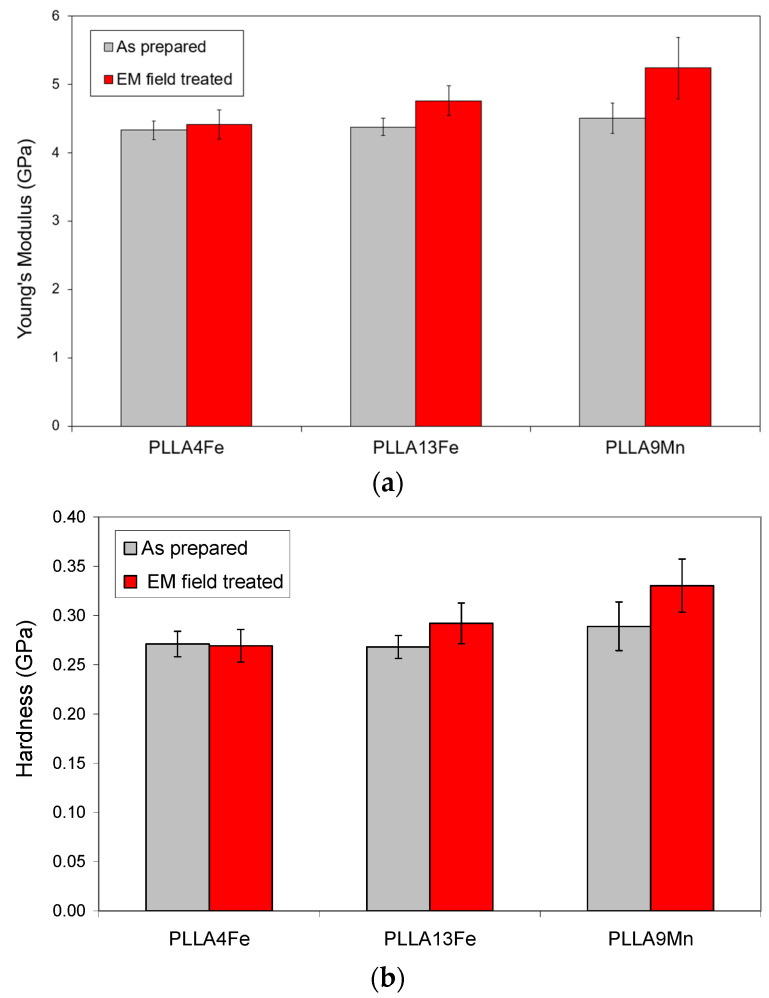
Mechanical properties of PLLA composites reinforced with Fe_2_O_3_ and MnFe_2_O_4_ MNPs, before and after EM field treatment: (**a**) Young´s Modulus and (**b**) Hardness.

The hardness and modulus average values of PLLA4Fe, PLLA13Fe, and PLLA9Mn composites before and after EM field treatments are presented in Figure 8. Indentations localized on the MNPs clusters were not considered.

Hardness and Young´s modulus are similar for all as-prepared composites as well as for the PLLA4Fe-331kHz sample.

The composites with high MNPs concentration treated by EMF have higher modulus and hardness values than those in the as-prepared condition. This increase of stiffness and hardness is greater in samples with MnFe_2_O_4_ MNPs. This effect could be related to the stabilization of the less stable crystallites caused by the EM field treatment, as the DSC curves show.

The modulus values obtained in the PLLA9Mn_112kHz composite are higher than those reported by Cifuentes et al. [28] for PLLA with higher crystallinity. This difference may be associated with the reinforcement with ceramic nanoparticles instead of Mg particles and the different testing conditions.

**Figure 9:**
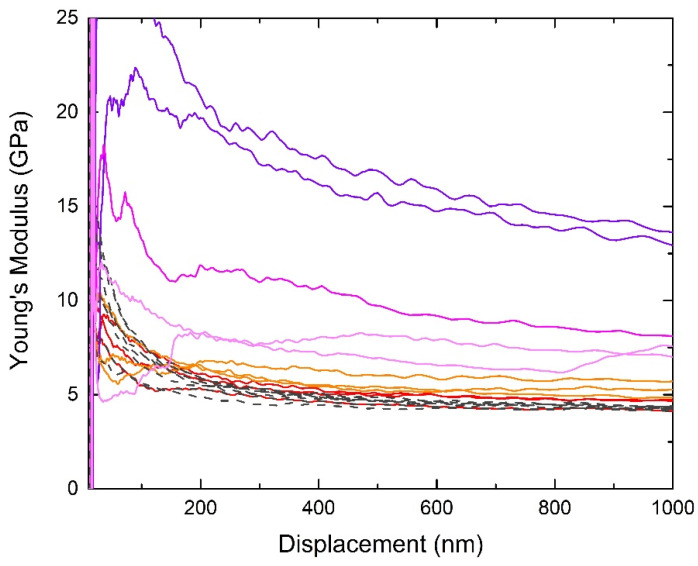
Modulus–displacement curves of different zones of PLLA9Mn_112 kHz composite: PLLA without heterogeneities (red and orange line), micrometric NPs clusters (purple line), and zones affected by smaller NPs aggregates (pink line). (Black dash line corresponding to PLLA9Mn.)

Figure 9 shows the hardness and modulus profile curves obtained in PLLA9Mn_112 kHz composites in different zones tested.

The indentations on PLLA with homogeneous dispersion of nanoparticles showed modulus values between 4.5 and 6.0 GPa (red and orange line) at depths in the range of 500–1000 nm. The same indentations on the PLLA9Mn as-prepared sample show lower modulus values and less dispersion (gray dashed line). The presence of stiffer zones in the PLLA of EM field-treated PLLA9Mn is in agreement with the stabilization of the less stable crystallites in those composites due to the effect of the electromagnetic field.

The curves with higher Young’s modulus correspond to micrometric clusters of nanoparticles (purple color). The modulus values of these areas were higher than 10 GPa in the range of 500–1000 nm. The indentations made on zones with smaller aggregated MNPs show modulus values intermediate between the matrix and micrometric cluster (pink line) within the range of 6–10 GPa. Both regions with aggregate MNPs were not considered for the determination of the modulus and hardness average values, as shown in Figure 2.

**Figure 10:**
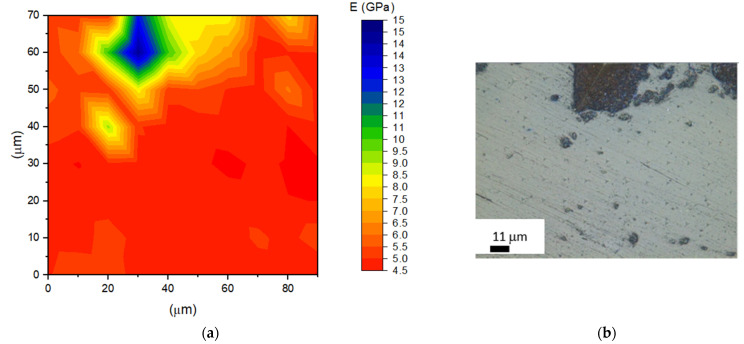
(**a**) Contour map showing the Young’s modulus distribution in PLLA9Mn as prepared; (**b**) OM picture of the area shown.

**Figure 11:**
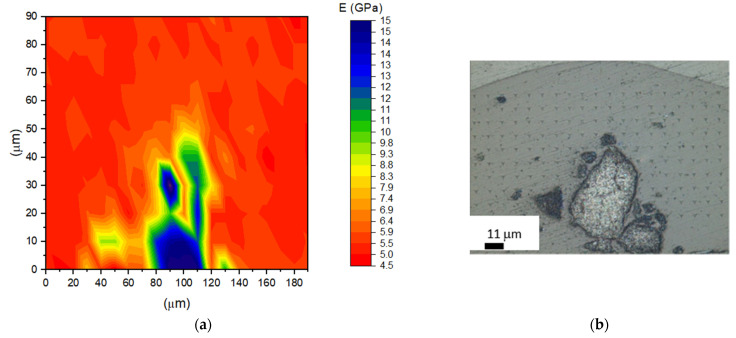
(**a**) Contour map showing the Young’s modulus distribution in EM field-treated PLLA9Mn; (**b**) OM picture of the area shown.

Figure 10 and Figure 11 represent the nanomodulus distribution in PLLA9Mn composites in the as-prepared state and after EM field treatment, as shown in the contour map (a) and the optic microscope (OM) picture (b). A comparison of both contour maps shows the variations of stiffness around the clusters for the EM field-treated sample in the zones of PLLA without heterogeneities. Stiffer areas can be observed in accordance with the stabilization of crystalline regions associated with MNPs’ heating effect.

Furthermore, the differences in the residual depth of the indents can be observed, providing evidence of variance in the recovery and visco-elastic behavior of both materials.

## 4. Conclusions

The crystallinity of composite materials made of PLLA matrix with MNPs is different from that of unreinforced materials, indicating that MNPs tend to modify the PLLA structure, thus limiting the formation of crystalline structures.

The crystallinity degree, hardness, and elastic modulus of composite materials made of PLLA matrix reinforced with certain magnetic nanoparticle concentrations was modified by using radiofrequency electromagnetic fields, as proved through calorimetric and nanoindentation characterization. Those fields induce heat in the MNPs, which spreads to the matrix, allowing changes in the order of the polymeric chains. However, to better comprehend the relationship between the magnetic and heating properties of MNPs and PLLA structure, the dispersion of MNPs inside the polymeric matrix should be improved.

The obtained results suggest that the modulation of an implanted biomaterial’s properties could be undertaken from the outside of the body in a noninvasive way and within the biological limit range *f⋅H* < 5 × 10^9^ Am^−1^·s^−1^.

## Figures and Tables

**Table 1:** MNPs concentration and Temperature of maximum weight loss rate, *T_p_*, of composites obtained from TGA results.

Sample	[MNPs] (wt%)	*T_p_* (°C)
PLLA4Fe	4.1	337
PLLA13Fe	12.7	324
PLLA2Mn	1.9	313
PLLA7Mn	7.4	301
PLLA9Mn	8.7	290

**Table 2:** MNPs material, wt% of MNPs in water, frequency (*f*), magnetic field amplitude (*H*), specific absorption rate (SAR), and dT/dt slope.

MNPs	wt%	*f* (kHz)	*H* (kAm^−1^)	SAR (W/g)	d*T*/d*t* (K/s)
γ-Fe_2_O_3_	13.4	112	18.5	6	0.20
		331	10.1	17	0.55
MnFe_2_O_4_	1.5	112	18.5	117	0.42
		331	10.1	36	0.13

**Table 3:** Composite material, wt% of MNPs in PLLA, frequency (*f*), magnetic field amplitude (*H*), specific absorption rate (SAR), initial temperature (*T_o_*), temperature after 600 s of EM field treatment (*T*_600s_), temperature increment (Δ*T*).

Composite	wt%	f (kHz)	*H* (kAm^−1^)	SAR (W/g)	*T_o_* (°C)	*T_600s_* (°C)	∆*T* (°C)
PLLA4Fe	4.1	331	10.1	0.4	25.2	27.7	2.5
PLLA13Fe	12.7	112	18.5	0.5	26.6	31.0	4.4
PLLA13Fe	12.7	331	10.1	0.7	24.8	30.1	5.3
PLLA7Mn	7.4	331	10.1	0.7	23.6	29.5	5.9
PLLA9Mn	8.7	112	18.5	4.3	21.8	35.5	13.7
PLLA9Mn	8.7	331	10.1	1.1	24.1	30.2	6.1

**Table 4:** Glass transition temperature, *T*_g_ (±1 °C), cold crystallization temperature, *T*_cc_ (±1 °C), crystalline fraction, *f*_c_ (±4%), and melting temperatures, *T*_m1_ and *T*_m2_ (±1 °C).

Material	*T_g_* (°C)	*T_cc_* (°C)	*f_c_* (%)	*T_m1_* (°C)	*T_m2_* (°C)
PLLA	53	97	38		150
PLLA4Fe-AP	36	93	29	144	158
PLLA4Fe-331 kHz	39	93	28	144	156
PLLA13Fe-AP	35	91	34		150
PLLA13Fe-112 kHz	36	94	21		151
PLLA13Fe-331 kHz	37	94	35	143	168
PLLA7Mn-AP	41	94	31		150
PLLA7Mn-331 kHz	42	94	26		151
PLLA9Mn-AP	38	95	20	143	154
PLLA9Mn-112 kHz	37	95	21	136	146
PLLA9Mn-331 kHz	38	90	33		148

## Data Availability

The data that support this research are available from the corresponding author upon reasonable request.

## Supplementary Materials

The following are available online at https://www.mdpi.com/article/10.3390/ma14154300/s1. Figure S1: Heating curves of PLLA9Mn samples during the EMF treatment, recorded by infrared camera, Figure S2: Heating curves of PLLA13Fe samples during the EMF treatment, recorded by infrared camera, and Table S1: Representative bands of PLA and corresponding region in FTIR spectroscopy.
