# Supplementary material for: Modulation of Crystallinity through Radiofrequency Electromagnetic Fields in PLLA/Magnetic Nanoparticles Composites: A Proof of Concept
Source: Materials (Basel). 2021 Jul 31;14(15):4300. doi: 10.3390/ma14154300 (PMC8347328; doi:10.3390/ma14154300)
Supplement: Supplementary file 1 [file materials-14-04300-s001.zip › materials-1292703-supplementary.pdf]

materials

MDPI

Supplementary Materials

Modulation of Crystallinity through Radiofrequency Electromagnetic Fields in PLLA/Magnetic Nanoparticles Composites: A Proof of Concept

Marta Multigner [1,]*, Irene Morales [2], Marta Muñoz [1], Victoria Bonache [1], Fernando Giacomone [2], Patricia de la Presa [2], Rosario Benavente [3], Belén Torres [1], Diego Mantovani [4] and Joaquín Rams [1]

[1] Department of Applied Mathematics, Materials Science and Engineering and Electronic Technology, Universidad Rey Juan Carlos, 28933 Madrid, Spain; marta.munoz@urjc.es (M.M.); victoria.bonache@urjc.es (V.B.); belen.torres@urjc.es (B.T.); joaquin.rams@urjc.es (J.R.)

[2] Instituto de Magnetismo Aplicado, UCM-ADIF-CSIC, A6 22,500 Km, 28230 Las Rozas, Spain; irenemorales@ucm.es (I.M.); giacomone@gmx.es (F.G.); pmpresa@ucm.es (P.d.l.P.)

[3] Institute of Polymer Science and Technology (ICTP-CSIC), C/Juan de la Cierva 3, 28006 Madrid, Spain; rbenavente@ictp.csic.es

[4] Laboratory for Biomaterials & Bioengineering (CRC-I), Department Min-Met-Materials Engineering & Research Center CHU de Québec, Laval University, Québec, QC G1V 0A6, Canada; diego.mantovani@gmn.ulaval.ca

* Correspondence: marta.multigner@urjc.

Citation: Multigner, M.; Morales, I.; Muñoz, M.; Bonache, V.; Giacomone, F.; de la Presa, P.; Benavente, R.; Torres, B.; Mantovani, D.; Rams, J. Modulation of Crystallinity through Radiofrequency Electromagnetic Fields in PLLA/Magnetic Nanoparticles Composites: A Proof of Concept. *Materials* **2021**, *14*, 4300. https://doi.org/10.3390/ma14154300

Academic Editor: Hansang Kwon

Received: 24 June 2021
Accepted: 29 July 2021
Published: 31 July 2021

Publisher's Note: MDPI stays neutral with regard to jurisdictional claims in published maps and institutional affiliations.

Copyright: © 2021 by the authors. Licensee MDPI, Basel, Switzerland. This article is an open access article distributed under the terms and conditions of the Creative Commons Attribution (CC BY) license (http://creativecommons.org/licenses/by/4.0/).

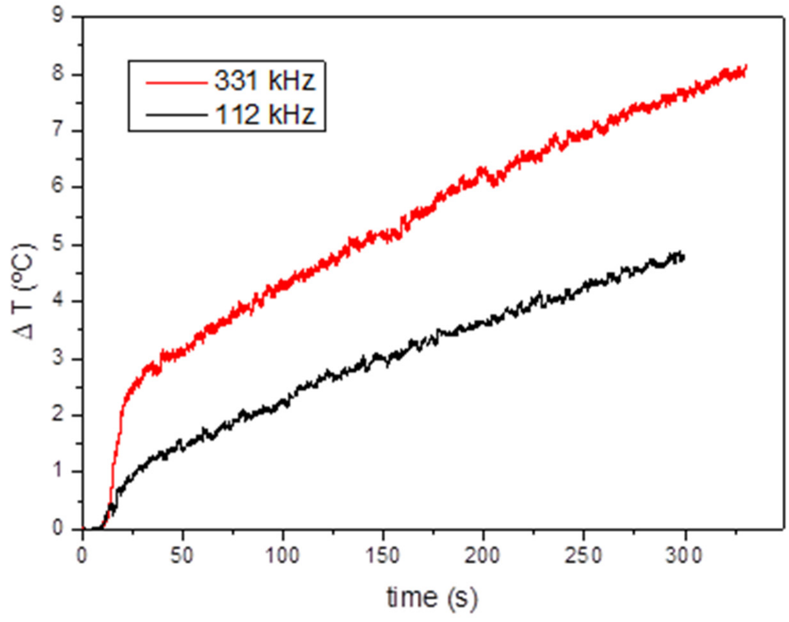

Figure S1. Heating curves of PLLA9Mn samples during the EMF treatment recorded by infrared camera.

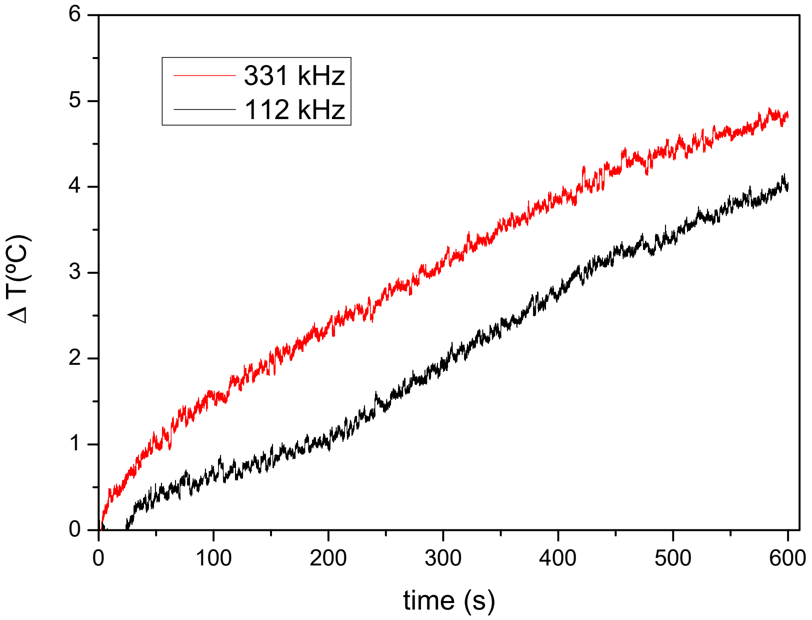

Figure S2. Heating curves of PLLA13Fe samples during the EMF treatment recorded by infrared camera.

Table S1. Representative bands of PLA and corresponding region in FTIR spectroscopy.

Region	Group or Bond
≥3000 cm^{-1} (missing)	–OH stretching vibration (carboxyl acids and alcohols)
3000–2800 cm^{-1}	CH region
1749 cm^{-1}	C=O stretching vibration of the carbonyl group
1456 cm^{-1}	Asymmetric bending of the CH_3
1361 cm^{-1}	Symmetric bending of the CH_3
1182 cm^{-1}	Asymmetric rocking of CH_3
1130 cm^{-1}	Symmetric rocking of CH_3
1083 cm^{-1}	Asymmetric stretching vibration of O–CO–O
1045 cm^{-1}	Stretching of C–CH_3
